# The Prevalence of Anaemia in a District General Hospital in the United Kingdom

**DOI:** 10.7759/cureus.15086

**Published:** 2021-05-18

**Authors:** Mohammed Hamid, Aysha Naz, Lakna H Alawattegama, Helen Steed

**Affiliations:** 1 General Surgery, University Hospital Birmingham National Health Service (NHS) Trust, Birmingham, GBR; 2 Endocrinology, Diabetes and Metabolism, Royal Wolverhampton Hospital Trust, Wolverhampton, GBR; 3 Orthopedics and Traumatology, Royal Wolverhampton Hospital Trust, Wolverhampton, GBR; 4 Gastroenterology and Hepatology, Royal Wolverhampton Hospital Trust, Wolverhampton, GBR

**Keywords:** anaemia, hospital, prevalence, 30 day mortality, 30 day readmission, general practice, biochemical investigation, falls, modifiable risk

## Abstract

Aim

Investigating the prevalence of hospital-acquired anaemia in a United Kingdom (UK) secondary care setting to describe the level of appropriate management prior to discharge back to primary care.

Design and settings

An observational study of 13 medical and surgical wards in a UK district general hospital.

Method

Single-day examination of notes, blood results and drug charts, with a 30-day follow up, using pre-set definitions of anaemia and exclusion criteria.

Results

Two hundred and sixty-seven patients were included. Of them, 52% were anaemic on admission, 62.2% were anaemic on the study day, 16% had hospital-acquired anaemia and 49%-82% had no biochemical indices checked during the admission or in the last 12 months. Also, 53% of anaemic patients are being discharged without appropriate treatment, with over a third being under-investigated.

Conclusion

The prevalence of anaemia in a UK district general hospital is high. Causes of anaemia are complex, posing a potentially modifiable risk factor for falls, readmission and mortality.

## Introduction

Anaemia is a common medical diagnosis, affecting 24.8% of the world’s population [1]. In the hospital, anaemia is associated with falls, higher mortality rates, and the consumption of additional resources, with an increased length of stay (LOS) and a shorter time to readmission [2,3]. Thirty-five million pounds in direct costs could be saved each year in the United Kingdom (UK) if anaemia management were to be optimised [4].

Patients admitted into hospitals are at risk of becoming anaemic from blood loss, nutritional issues or chronic disease [5]. American studies report that 30%-75% of patients admitted to secondary care will develop hospital-acquired anaemia (HAA), but there is a lack of data in the UK on this prevalence [6,7].

In New Zealand, a study on anaemia recognised that it was not well documented or investigated in the secondary care setting despite being a modifiable risk factor and a cancer diagnosis opportunity [2]. Although we rely on General Practitioners (GPs) to identify and characterise anaemia in the UK, managing anaemia in primary care has also been cited as a challenge; with under investigation remaining the predominant issue despite the availability of guidelines [8,9]. Given that some studies have demonstrated that almost half of elderly patients with an identified iron deficiency anaemia (IDA) have a gastrointestinal tract abnormality [10,11]; and with many anaemic patients being admitted having worse anaemia on discharge, there is a need to review UK practices in order to understand the current practice and identify areas for further research and improvement across the National Health Service (NHS).

The objective of this study was to determine the prevalence of anaemia and HAA in a UK secondary care setting and to describe the level of appropriate investigation and treatment taking place in hospital prior to patients being discharged back to their primary care physicians.

This article was previously presented as a meeting poster presentation at the 2019 Midlands Gastroenterology Society Annual Meeting on May 10, 2019.

## Materials and methods

The Royal Wolverhampton Trust - Foundation Trainee Research Group (RWT-FTRG) undertook an observational study across 13 wards present in New Cross, Wolverhampton - a UK district general hospital, on a single day in September 2018. The wards represented general medical wards with speciality links, mixed general surgical wards including Orthopaedic and ENT wards.

Definitions

The World Health Organization (WHO) criteria for anaemia were used and are shown in Table 1.

HAA was defined as anaemia directly attributable to hospitalisation; a reduction in haemoglobin (Hb) to anaemia levels during hospitalization with previously normal haemoglobin on admission blood.

Acute blood loss was defined as a loss of 500mL or more at the surgery or a documented endoscopically confirmed gastrointestinal bleed.

**Table 1:** World Health Organization (WHO) anaemia criteria for male and female patients.

Type of Anaemia	Male (g/dL)	Female (g/dL)
Mild	>11 to <13	>11 to <12
Moderate	>9 to <11	>9 to <11
Severe	<9	<9

Training

Two training sessions were held to cover inclusion and exclusion criteria; WHO anaemia criteria; other definitions; and the data recording tool. Foundation doctors performed the data collection in groups of two and three to reduce inter-rater variability; with real-time consultant-led supervision to resolve any queries the data collecting teams had. There were three queries raised suggesting good inter-rater concordance.

Validation

The supervising consultant subsequently reviewed a random case number from each data collecting team to ascertain data validity with no collection errors identified.

Data collection

Electronic laboratory records, inpatient notes and medication charts were reviewed to extract the relevant data. Demographic information was recorded, along with current anaemia status and severity, whether anaemia was present on admission blood, and any evidence of pathological or iatrogenic acute blood loss.

Subgroups identified as having HAA or acute blood loss were followed up until 30-day post-discharge for records of falls, 30-day mortality and 30-day readmission.

Inclusion criteria

Adult patients aged 18 years and over who had been admitted for more than 24 hours at the time of the study were included.

Exclusion criteria

Patients under 18 years old, patients on critical care, active haematology patients, pregnant or postpartum women, patients admitted for less than 24 hours, and patients with no recorded haemoglobin.

## Results

Demographics

A total of 267 patients were included. Men made up 46% of patients with a mean age of 70 (range 23-98), women had a mean age of 67 (range 18-100). The patients had spent a total of 4,207 days in the hospital, with a mean LOS of 16 days (range 1-232).

Seventy-five per cent had one or more significant comorbidities of the major bodily systems including cardiac, renal, liver, respiratory, cancer, stroke or other serious conditions as shown in Table 2.

**Table 2:** Comorbidities of the total cohort and the subgroup of anaemic patients. IHD: Ischaemic heart disease, AF: Atrial fibrillation, SVD: Severe valve disease, CCF: Congestive cardiac failure, CKD: Chronic kidney disease, COPD: Chronic obstructive pulmonary disease.

Comorbidities	All (n=267)	Anaemia (n=166)
None	68	34
Cardiac (IHD, AF, SVD, CCF)	131	67
Renal impairment (CKD 3/4)	36	24
Liver (Cirrhosis)	19	14
Respiratory (COPD)	40	24
Active Cancer	22	1
Stroke	31	18
Other serious comorbidities	100	58

Anaemia

**Figure 1:**
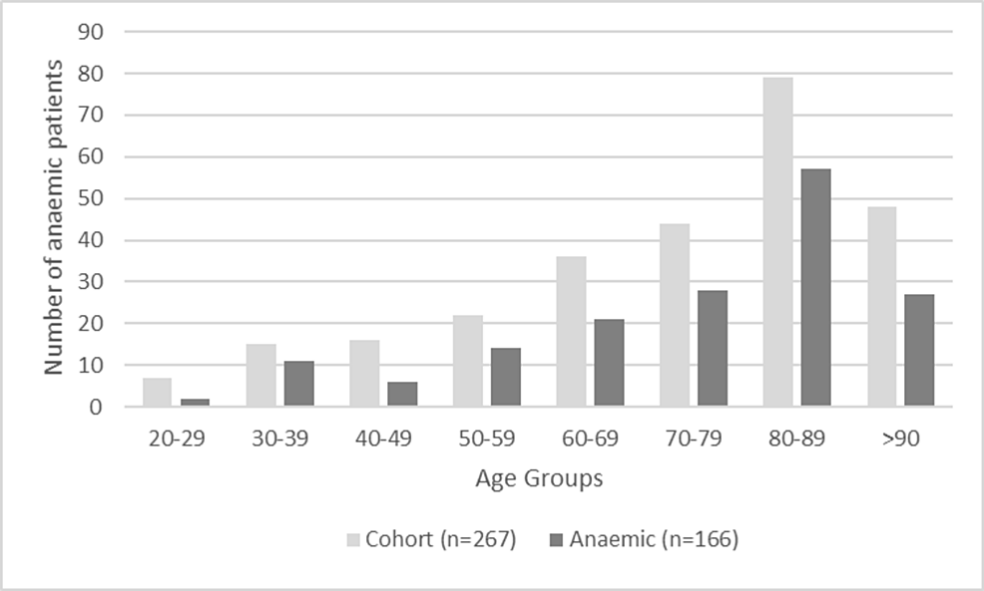
The number of anaemic patients per age group in relation to the whole cohort studied.

Fifty-two per cent of patients were anaemic on admission (54% were male, 46% were female and 3% were females under 45 years old). On the study date, 62% were anaemic, of these 35% had mild anaemia, 35% moderate anaemia and 30% severe anaemia. Sixty-seven per cent of the patient’s over 65 years old were anaemic on the study day, increasing from 56% who had been anaemic on admission. Figure 1 demonstrates the number of anaemic patients relative to the whole cohort, clustered into age groups.

**Figure 2:**
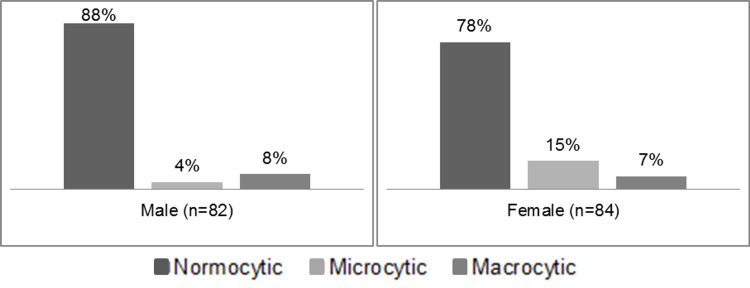
Comparing the types of anaemia across the anaemic male and female patients.

Comparing the types of anaemia as a percentage in all patients studied (n=267), 33% had normal Hb, 56% had normocytic anaemia, 6% microcytic and 5% macrocytic anaemia. Figure 2 shows charts for anaemic male and anaemic female patients.

Investigations

During their current admission, 6% of patients had all haematinics investigated, 11% within the last 12 months. Six (4%) of all anaemic patients had complete investigations including C-reactive protein (CRP) and myeloma screen. Forty-eight (32%) of normocytic patients had vitamin B12, folate and ferritin checked; eight (47%) of microcytic patients had B12 and folate checked and nine (75%) of macrocytic patients had ferritin checked on admission or within the last 12 months. Table 3 shows whether patients had any biochemical investigations performed in relation to anaemia at the time of the study, irrespective of LOS at that point.

**Table 3:** Level of investigation for anaemic patients. The percentage represents the quantity of anaemic patients investigated for vitamin B12, folate, ferritin, serum iron levels, CRP and myeloma screen (n=166). CRP: C-reactive protein

Investigations	Not checked	Checked on admission	Checked in last year	Appropriately not checked
B12	41%	20%	33%	6%
Folate	42%	19%	33%	6%
Ferritin	52%	16%	26%	6%
Serum Iron	81%	6%	7%	6%
CRP	7%	93%	-	-
Myeloma screen	70%	17%	-	13%

Treatment

On the day of the study, sub-analysis of the anaemic patients' (n=166) regular medication charts, 20% were on or had received oral or intravenous iron, 22% on folic acid and 4% on oral or intramuscular B12.

Discharge

As there may have been medical reasons for not biochemically investigating patients at the stage of admission as captured by the study, patients being discharged were analysed as a subgroup. On the day of the study, 19% of patients (n=51) were discharged; and of these 23 were anaemic (0 macrocytic, 3 microcytic, 20 normocytic). Of the 23 anaemic patients, 10 (43%) were discharged on the appropriate vitamin replacement medication (i.e. B12 and folate if macrocytic, iron if microcytic). Eight (35%) of the anaemic patients remained biochemically un-investigated.

Hospital-acquired anaemia

Sixteen per cent (n=43) of inpatients had HAA, which represented 25% of the anaemic patients on the day of the study. Ninety-seven per cent of this group were still anaemic when later discharged, and 27% were on or had received appropriate vitamin replacement medication, or did not require medication after appropriate investigations at the time of discharge. The 30-day mortality was 2%, the 30-day readmission rate was 18% and 3% had documented falls with no other identifiable cause found besides anaemia.

Acute blood loss

Thirteen per cent of patients in the study had acute blood loss (n=35), almost half of whom were anaemic on admission. These 35 patients contributed to 21% of the overall anaemia cases in the group and equate to 42% of the HAA cases. Three acute blood loss patients died during admission. Ninety-six per cent were anaemic on discharge and 44% on treatment for anaemia at discharge. There was 3% 30-day mortality, 23% 30-day readmission rate and 6% had a documented fall.

## Discussion

Strengths and limitations

Our study was limited by the relatively small sample size, but the results are matched by similarly sized and much larger studies in other countries as discussed below.

We recognise that the aetiology of anaemia is complex and multifactorial. Low haemoglobin levels may represent a de novo diagnosis, a chronic status, the result of an acute illness, or the cumulative effect of several other confounding factors, not fully addressed in this study. All the aforementioned facets challenge any correlations made with hospitalisation and readmission.

The level of biochemical investigations at the time of the study was a snapshot in the patient’s admission, and a limitation of the study is that some or all patients may have potentially been investigated later in their admission. However, 19% of patients discharged on the day of the study offer a possible reflection of what may occur and indicate that a proportionate percentage of patients were not investigated by the time of discharge.

The reasons behind under-investigation are not explored in this study and could potentially include: causes of anaemia are complex; anaemia is so commonplace it is overlooked or dismissed as an expected finding in someone with multiple comorbidities; circumstances within an admission are dynamic, e.g. ongoing active bleeding or inappropriate investigation as the patient is actively dying.

Comparison with existing literature

Results are similar to those reported in other European countries, the US and a large systematic review [6, 12-17]. An Italian observational study of 923 consecutive admissions of 856 patients found a prevalence of anaemia of 58.4% [14]. Also, a German cross-sectional study reported a prevalence of anaemia of 60% in 100 elderly inpatients over 70 years old [18]. A systematic review of the prevalence of anaemia in older persons in 2008 found 45 studies to contribute to the analysis and reported an anaemia prevalence of 40%-72% of hospital admissions for the patient over the age of 65 [19].

Implications for research and/or practice

The management of anaemia and its associated comorbidities is a shared endeavour between primary and secondary care. In the UK, the NHS relies on GPs to identify, characterise and initiate treatment or referral for further investigation of anaemia. When patients are admitted into a hospital with an acute non-related illness such as community-acquired pneumonia, the cost-benefit margin of keeping patients in to investigate a mildly abnormal or asymptomatic haemoglobin level is unfavourable; and also, less practical as laboratory results often return after the patient has been discharged. Ultimately, each hospital admission episode results in anaemic patients being sent home in a worse state, with not enough being done in the secondary care contact point to address anaemia.

There exists an unstructured communication portal between hospitals and GPs regarding the follow up of anaemic patients, which may contribute to the delayed diagnosis of potentially life-altering conditions.

IDA can be a sign of gastrointestinal cancer. In the older adult inpatient population, IDA has been shown to contribute to 15%-30% of anaemia cases [20]. Therefore, failure to recognise iron deficiency as a cause of normocytic as well as microcytic anaemia in this population represents a potential missed opportunity to diagnose malignancy [21].

Equally, over-treatment of anaemia with inappropriate iron could have unintended consequences, particularly in the elderly. Side effects can affect up to 20% of patients treated with oral iron, including nausea and diarrhoea and change of appetite [22].

Some guidelines advocate that patients who are anaemic on a full-blood count warrant further investigation; including B12 and folate, iron studies, ferritin, and renal or liver function tests [19]. We have initiated a quality improvement project to investigate the impact of an anaemia hospital discharge prompt, similar to the acute kidney injury (AKI) prompts currently utilised by hospitals across the UK. The prompt would provide a communication bridge between secondary and primary care to flag the haemoglobin level and advise the further steps required of the GP to follow up when the patient returns to the community.

## Conclusions

This study confirms that anaemia is highly prevalent in the population being admitted to a UK district general hospital and that an inpatient stay represents a substantial risk for HAA. The prevalence of anaemia in a UK district general hospital is high and comparable to published data worldwide. The causes of both chronic anaemia and hospital-acquired anaemia are complex, but anaemia poses a potentially modifiable risk factor for falls, 30-day readmission and mortality not being adequately addressed.

Further work is needed to identify if aggressive investigation and treatment of anaemia, where appropriate in the hospital, will alter the outcomes for falls, readmission and mortality, and whether the use of a simple communication bridge between secondary and primary care regarding the discharge of anaemic patients will impact the overall prevalence of anaemia and reduce the time to gastrointestinal cancer investigation.
